# Safe uses of Hill's model: an exact comparison with the Adair-Klotz model

**DOI:** 10.1186/1742-4682-8-10

**Published:** 2011-04-26

**Authors:** Zoran Konkoli

**Affiliations:** 1 Chalmers University of Technology, Department of Microtechnology and Nanoscience, Bionano Systems Laboratory, Sweden

## Abstract

**Background:**

The Hill function and the related Hill model are used frequently to study processes in the living cell. There are very few studies investigating the situations in which the model can be safely used. For example, it has been shown, at the mean field level, that the dose response curve obtained from a Hill model agrees well with the dose response curves obtained from a more complicated Adair-Klotz model, provided that the parameters of the Adair-Klotz model describe strongly cooperative binding. However, it has not been established whether such findings can be extended to other properties and non-mean field (stochastic) versions of the same, or other, models.

**Results:**

In this work a rather generic quantitative framework for approaching such a problem is suggested. The main idea is to focus on comparing the particle number distribution functions for Hill's and Adair-Klotz's models instead of investigating a particular property (e.g. the dose response curve). The approach is valid for any model that can be mathematically related to the Hill model. The Adair-Klotz model is used to illustrate the technique. One main and two auxiliary similarity measures were introduced to compare the distributions in a quantitative way. Both time dependent and the equilibrium properties of the similarity measures were studied.

**Conclusions:**

A strongly cooperative Adair-Klotz model can be replaced by a suitable Hill model in such a way that any property computed from the two models, even the one describing stochastic features, is approximately the same. The quantitative analysis showed that boundaries of the regions in the parameter space where the models behave in the same way exhibit a rather rich structure.

## Background

The Hill function and the related Hill model [1] are used frequently to study biochemical processes in the living cell. In strict chemical terms Hill's model is defined as(1)

where *C *denotes a protein that binds ligands, *A *is a ligand, and *C_h _*is a ligand-protein complex having *hA *molecules attached to *C*. The stoichiometric coefficient *h *describes the number of ligand binding sites on the protein. All ligands bind at once. Both the forward and the back reactions are allowed. It is relatively simple to derive the expression for the dose response curve (the Hill function) which relates the amount of free ligands, *a*, to the fraction of ligand-bound proteins (e.g. receptors) in the system, *φ*. The Hill function is given by(2)

where *K*_0 _denotes the dissociation constant.

The Hill function is used frequently in various areas of physics, biology, and chemistry. For example, it is widely used in pharmacological modeling [2], as well as in the modeling of biochemical networks [3]. In the most common scenario, the Hill function is fitted to an experimentally obtained dose response curve to infer the value of the stoichiometry coefficient, *h*. The value obtained in such a way is not necessarily an integer number and is referred to as the Hill coefficient. The number of ligand binding sites is an upper limit for the Hill coefficient. The Hill coefficient would reach this limit only in the case of very strong cooperativity. More discussions on the topic can be found in [4]. However, in present study, the variable *h *will be allowed only non-negative integer values.

Hill's model has been heavily criticized since it describes a situation where all ligands bind in one step [5]. In reality, simultaneous binding of many ligands is a very unlikely event. A series of alternative models have been suggested where such assumption is not implicit [6-8]. A typical example is the Adair-Klotz model [6] defined as(3)(4)

with *i *= 1, ..., *h'*. Protein *C *binds ligands successively in *h*' steps. Here, and in the following, the subscript *i *on *C *denotes the number of *A *molecules attached to it, with the obvious definition *C*_0 _≡ *C*. Apparently, in comparison to the Hill model, the alternative models - while being more realistic - are more complicated and harder to deal with (e.g. the Adair-Klotz model shown above). Accordingly, the central question being addressed in this work is whether it is possible to establish conditions where Hill's model can be used safely as a substitute for a more complicated reaction model. With a generic understanding of when this can be done, it should be possible to study an arbitrary reaction system with the elegance that comes with the use of Hill's model, knowing at the same time that the results are accurate. Also, even if there is evidence that the Hill model might describe the problem, it is not immediately clear which features of the problem can be described faithfully.

In the following, Hill's model will be compared with a well chosen reaction model that is more realistic, and not too complicated from the technical point of view. The Adair-Klotz model discussed previously is a natural choice since it assumes that ligands bind sequentially, and the model is relatively simple to deal with.

Furthermore, it is necessary to choose which property to study. For example, Hill's and Adair-Klotz's models have been compared in [5] where the property of interest was the dose-response curve *φ*(*a*). Using classical chemical kinetics, the dose-response curves predicted from Adair-Klotz's and Hill's model were compared neglecting fluctuations in particle numbers. It was found that for a strongly cooperative Adair-Klotz model it is possible to find the parameters for Hill's model that will result in similar dose response curves. The question is what happens for other properties, and what happens when fluctuations in particle numbers are taken into account?

To avoid dealing with a particular choice of a property of interest, and to strive for an exact treatment, the models will be compared on the level of the respective particle number distributions. The position developed in this work is that the particle number distribution function of a model is the fundamental quantity that describes all features of the system. If the particle number distributions are similar, any property computed from them should have numerical values that are close. For example, the relevant variable for both models is the number of free ligands in the system. If the particle number distribution functions are same for both models then the resulting number of free ligands will be same. However, the opposite might not hold: it might be that the number of free ligands is same but some other quantity (e.g. fluctuations in the number of free ligands) might be be vastly different. To avoid such traps, the focus is on comparing the particle number distribution functions directly.

The scope of the analysis in [5] will be extended in several ways. First, in addition to studying the stationary (equilibrium) properties of the models, dynamics will be studied as well. Many processes in the cell are strongly time dependent and involve cooperative binding, such as the early stages of signalling processes, and cascades in later stages of signal propagation phase. Likewise, many processes in the cell need to happen in a particular order. Clearly, the time and dynamics play a crucial role in the workings of cell biochemistry. Second, the previous mean field (classical kinetics) analysis will be extended to account for effects of fluctuations (intrinsic noise). It has been recognized that intrinsic noise (fluctuations in the numbers of particles) is not just a nuisance that the cell has to deal with, but is an important mechanism used by the cell to function [9-12]. Intrinsic noise becomes important when protein copy numbers are low. Such a situation is frequent in the cell (e.g. gene expression networks). Third, a generic comparison of the models will be provided by focussing on the particle number distribution functions.

## Results and discussion

### Description of models

The models are parameterized as follows. Hill's model is parameterized by two reaction rates for the forward and the back reactions that will be denoted by *α *and *β *respectively. The dissociation constant for the model *K*_0 _is governed by the ratio *β/α *and for simplicity it will be assumed that(5)

The Adair-Klotz model involves more parameters: the forward and the back reaction rates for an *i*-th reaction are given by *α_i _*and *β_i _*respectively, and *i *= 1, ..., *h*'. The dissociation constants for the Adair-Klotz model are defined as(6)

It is assumed that the particles mix well and that it is sufficient to count the particles. The models are stochastic and are described using the continuous time Markov chain formalism [13]. The reaction rates govern the transition probabilities between states of the system. The master equations for the models are the consequence of the corresponding forward Chapman-Kolmogorov equations for the transition probabilities. The solutions of the master equations are the particle number distribution functions as explained in the "Computation of the distribution functions" section. To compare the distribution functions for the models, three similarity measures are defined in the "Comparison of the distribution functions" and "Fine tuning the comparison procedure" sections.

From the model-centric view taken in this investigation, the best way to compare the distribution functions is to choose *h *= *h*'. This makes the number of binding steps in the Adair-Klotz model equal to the stoichiometric coefficient of the Hill model. Also, within the scope of this work, to simplify wording, the variable *h *will be simply referred to as the Hill coefficient. The choice *h *= *h*' makes it possible to relate the distribution functions in a rather natural way. Namely, if *h *= *h*', it is possible to establish a one to one correspondence between Hill's model state space and a subspace of Adair-Klotz's model state space. The respective states in these spaces will be referred to as common states, or the common state space.

The first similarity measure defined, *δ*(*t*), quantifies the similarity between the distribution functions for Hill's and Adair-Klotz's models on the space of common states. In the text this similarity measure is referred to as the main or fundamental similarity measure. The states in Adair-Klotz's model state space that are not part of the common state space are referred to as the complement (state) space. This set contains states in which at least one of the intermediate species (section "Computation of the distribution functions") is present. These states are unique to Adair-Klotz's model.

The second similarity measure introduced , measures the extent to which the complement space is occupied. This is an auxiliary similarity measure that complements the information conveyed by the use of the fundamental similarity measure *δ*(*t*).

The third similarity measure, ,) quantifies the similarity between the shapes of Hill's and Adair-Klotz's model distribution functions. It is also an auxiliary similarity measure used to refine the information provided by inspection of the fundamental similarity measure. To compare the shapes of the distribution functions, Adair-Klotz's model distribution function is re-normalized on the common state space.

### Optimization of Hill's model parameters

One needs to be careful not to compare an arbitrary Hill's model to an arbitrary Adair-Klotz's model. Since the goal is to quantify which Adair-Klotz's models can be replaced by the related Hill's models, it is natural to choose the best possible parameters for the Hill model that maximize the fundamental similarity measure *δ*(*t*). Thus for each choice of the parameters for the Adair-Klotz model, the parameters of the Hill model will be optimized. The optimization procedure differs somewhat for plots that depict time dependence from the ones that depict equilibrium properties.

In the equilibrium, *δ*(*t*) depends only on the values of the dissociation constants: *δ*_∞ _= lim_*t*→∞ _*δ*(*t*) and(7)

For a fixed tuple (*K*_1_, *K*_2_, ..., *K_h_*) the Hill model dissociation constant *K*_0 _is optimized to make *δ_∞ _*as large as possible. This makes the Hill's model dissociation constant dependent on Adair-Klotz's model dissociation constants in a well defined way:(8)

where *g *is the function resulting from the optimization procedure. Thus one can write *δ_∞ _*= *f(g(K*_1_, *K*_2_, ..., *K_h_*), *K*_1_, *K*_2_, ..., *K_h_*), which defines the function *δ*_max _such that for a given choice of dissociation constants for the Adair-Klotz model *δ*_∞ _is the largest possible(9)

The function *δ*_max _is depicted in all plots that analyze the equilibrium state.

Please note that the use of Eq. (8) only fixes the ratio *β/α*. Accordingly, for time dependent plots, an additional choice has to be made for either *α *or *β*. For a time dependent plot the value for *α *was adjusted so as to make the life-time of the initial state the same in both models. (During the optimization, the value of *β *is given by *K*_0_*α*).

### Numerical results

**Figure 1:**
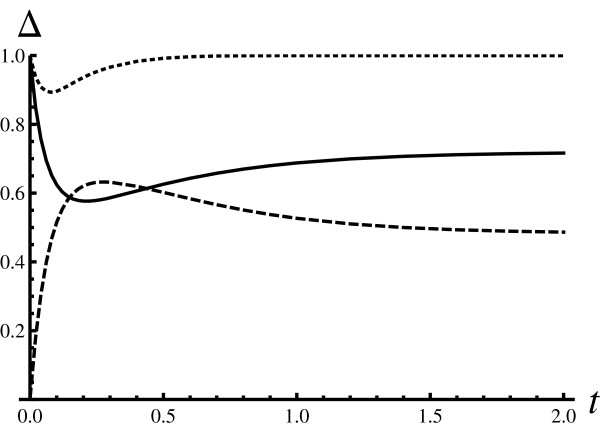
**Similarity measures (weakly cooperative Adair-Klotz model, *h *= 2)**. Time dependence of the similarity measures for *h *= 2 case: . This and all other figures in the manuscript were generated with *P*_0 _= 2 and *L*_0 _= 5. In this figure weakly cooperative Adair-Klotz model has been considered with *α_i _*= *β_i _*= 1*s*^-1 ^for *i *= 1, ..., *h*. The parameters for the Hill model were optimized so that *δ*(*t*) is largest possible (*β*/*α *= 0.5 and *α *= 0.5*s*^-1^). The time *t *is expressed in units of *s*. The full line is for Δ = *δ*, while the dashed and the dotted lines are for  and  respectively.

The three similarity measures have been computed numerically by solving the master equations for the models. Figure 1 shows how the similarity measures  depend on time in the situation where it is expected that Hill's model cannot approximate the dynamics of Adair-Klotz's model, i.e. when all reaction rates are equal and Adair-Klotz's reaction system cannot be described as cooperative. The similarity is perfect at *t *= 0 by construction, since in principle both systems are prepared in identical states. The similarity starts decreasing since the intermediate states become populated. This can be seen from the fact that the dashed line goes up, starting from zero. Please note that after some time the intermediate states become de-populated since the dashed line goes down after the initial peak around *t *≈ 0.25. The choice of reaction rates for the Adair-Klotz model clearly makes the intermediate states long lived. In such a case it is not possible to find the parameters *α *and *β *such that the fundamental (main) similarity measure is large.

The first auxiliary similarity measure that relates the shapes of the distribution functions (the dotted line in the figure) exhibits interesting behaviour:  for all times (early, intermediate, and asymptotic). Given this insight, one can conclude that only properties (observables) that are shape sensitive can be described by Hill's model, despite the fact that intermediate states are highly populated. For example, the moments of the particle number distributions do not fall into this category (e.g. the average numbers of particles in the systems or the variances); however, ratios of moments (defined on the common state space) do.

**Figure 2:**
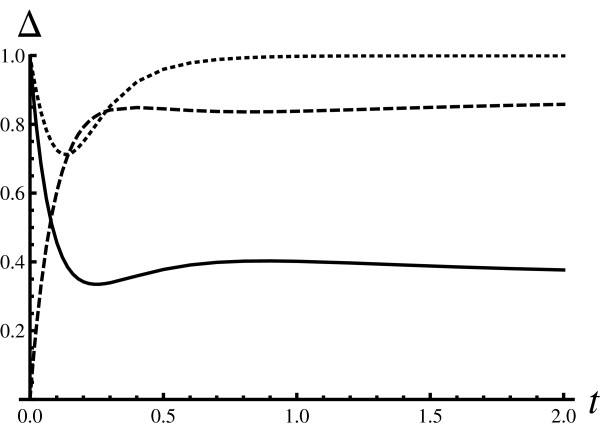
**Similarity measures (weakly cooperative Adair-Klotz model, *h *= 3)**. Generated in the same way as Figure 1, but with a higher value for the Hill coefficient (*h *= 3). The parameters of the Hill model were optimized in the same way as for Figure 1, resulting in *α *= 0.5*s*^-1^, *β *= 0.083*s*^-1^. Increase in the Hill coefficient makes the discrepancy larger since there are more intermediate states that can be populated. The similarity in the distributions shape increases for large times.

To which extent are the findings discussed so far sensitive to the value of the Hill coefficient? Figure 2 was constructed in the same way as Figure 1, but with a higher value of the Hill coefficient. To make the computations faster, the lowest possible value for the Hill coefficient was used, i.e. *h *= 3. In comparison to the *h *= 2 case, the fundamental similarity measure decreases further. It can be seen that  increases, which indicates that the complement space becomes more populated. It is very likely that this is because more intermediate states are available. The shape similarity measure  decreases for intermediate times, as the dotted curve has a deeper minimum than the dotted curve in Figure 1.

**Figure 3:**
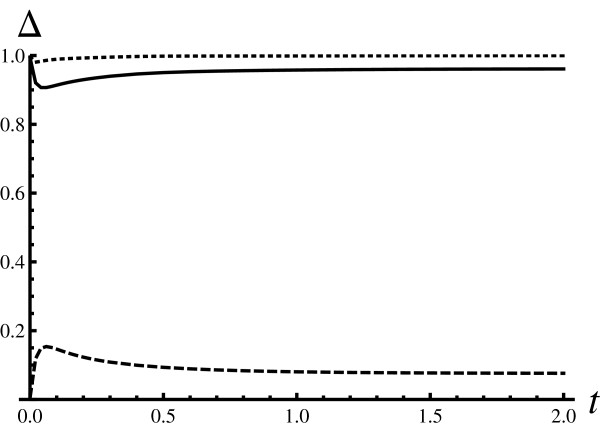
**Similarity measures (strongly cooperative Adair-Klotz model, *h *= 2)**. Generated in the same way as Figure 1, but with different values for the reaction rates. The particular choice of the reaction rates makes the intermediate states weakly populated: *α*_1 _= 1*s*^-1^, *β*_1 _= 10*s*^-1^, *α*_2 _= 10*s*^-1^, and *β*_2 _= 1*s*^-1^. The parameters for the Hill model were optimized in the same way as for the Figure 1 resulting in *α *= 0.5*s*^-1 ^and *β *= 0.25*s*^-1^. *δ*(*t*) stays relatively close to one indicating a good match. The dashed curve stays low, which indicates that intermediate states are short lived. The dotted line stays close to one indicating that the distributions have a similar shape.

For the case in which intermediate states are short lived, one intuitively expects that Hill's model could be a useful substitute for Adair-Klotz's model. Figure 3 depicts the dependence of the similarity measures on time, for systems that are expected to behave in a similar way. In particular, the reaction rates for the Adair-Klotz model used were chosen in such a way that the intermediate states are short lived. Indeed, the value of  stays very close to 0. The shapes similarity measure  stays very close to one, finally leading to large values for the fundamental similarity measure *δ*(*t*). This is an important finding since it indicates that Hill's model can be used to investigate an arbitrary observable, e.g., not just the average number of free ligands, but also the noise characteristics of that quantity. Naturally, such a claim comes with the implicit constraint that the observable should be interpreted in the context of Hill's model state space. For example, quantities such as the number of free receptor proteins, or the number of fully occupied receptors, fall in this category. However, any quantity that would involve counting the number of intermediates does not.

The time dependence of the similarity measures was investigated to confirm that these analysis tools work as expected. It is important to check that the analysis will work for both dynamics and the equilibrium state. In the following, the focus is on understanding equilibrium properties. The goal is systematically to identify situations when Hill's and Adair-Klotz's model distribution functions are similar. Technically, this will be done by mapping out regions in the Adair-Klot's model parameter space where the fundamental similarity measure *δ*_max _is relatively high.

**Figure 4:**
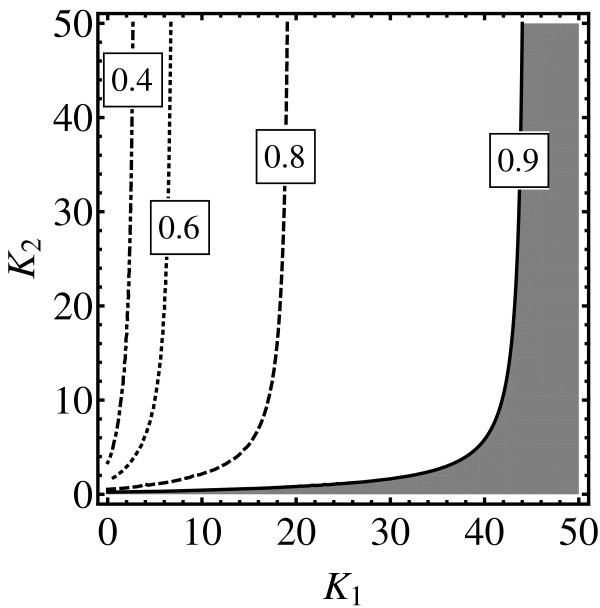
**Equilibrium state similarity measure for *h *= 2**. The contour plot that depicts how long time limit of *δ*_∞ _= lim_*t*→∞ _*δ*(*t*) depends on the dissociation constants *K*_1 _= *β*_1_/*α*_1 _and *K*_2 _= *β*_2_/*α*_2_; *δ*_∞ _= *f*(*K*_0_, *K*_1_, *K*_2_). For a fixed pair (*K*_1_, *K*_2_) the Hill model dissociation constant *K*_0 _= *β*/*α *is optimized to make *δ*_∞ _as large as possible, making the Hill's model dissociation constant dependent on Adair-Klotz's model dissociation constants in a well defined way; *K*_0 _= *g*(*K*_1_, *K*_2_) leading to the function *δ*_∞ _= *f*(*g*(*K*_1_, *K*_2_), *K*_1_, *K*_2_) = *δ*_max_(*K*_1_, *K*_2_) that is depicted in the plot.

Figure 4 shows how *δ*_max _depends on the values of the Adair-Klotz model reaction rates for the case *h *= 2. The figure depicts contours where *δ*_max _= *const *in the (*K*_1_, *K*_2_) plane. The first interesting region is in the range 0 ≤ *K*_1 _≲ 45 and below the full curve. In this range (the grey region below the full curve) *K*_1 _≫ *K*_2 _guarantees high similarity measure values. This analysis confirms the previous mean field study [5] where it was shown that choosing *K*_1 _≫ *K*_2 _leads to similar dose response curves. In the present article it has been shown that the results holds for any observable (average numbers, variances, etc). The second interesting region is for *K*_1 _≳ 45. In that region the fundamental similarity measure is large for any *K*_2_. Cases with relatively large values of *K*_2 _are not interesting chemically, since such reactions would be chemically non-functional: *K*_1_*K*_2 _≫ 1 would lead to the situation where the fraction of final products (complexes) in the system would be vanishingly small. However, a reaction with *K*_1 _≳ 45 and *K*_2 _≪ 1 could be functional provided *K*_1_*K*_2 _~1.

**Figure 5:**
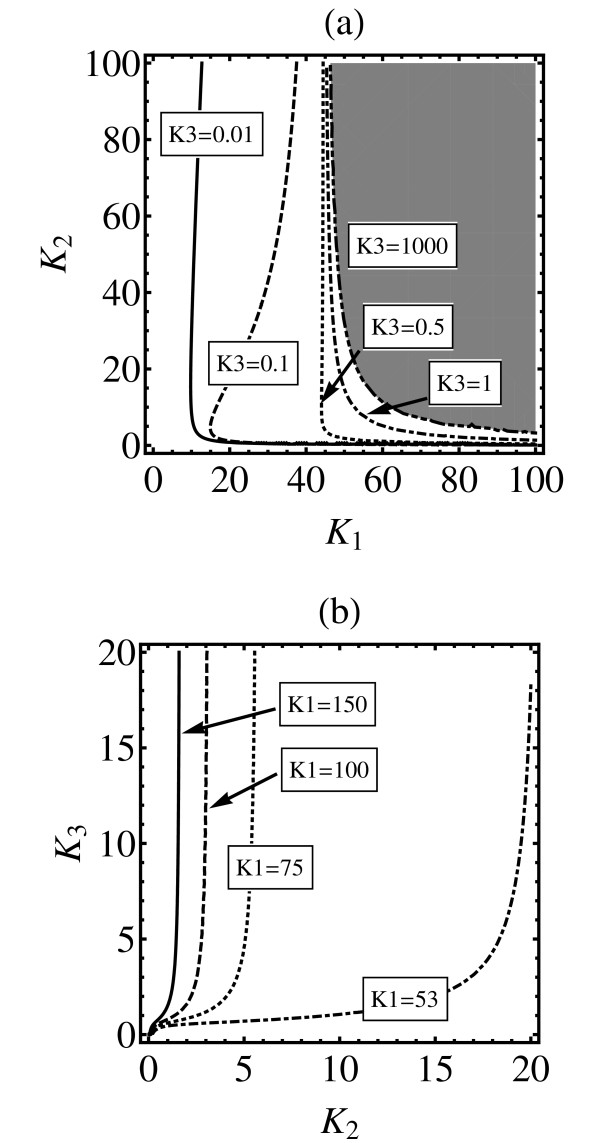
**Equilibrium state similarity measure for *h *= 3**. The plot depicts equilibrium state similarity measure for *h *= 3 case. For each triple (*K*_1_, *K*_2_, *K*_3_) an optimal value is found for *K*_0 _that maximizes *δ*_∞_. In such a way *δ*_∞ _= *δ*_max_(*K*_1_, *K*_2_, *K*_3_). The lines plotted in both panels denote the *δ*_∞ _= 0.9 boundaries. For a given curve, the region with *δ*_∞ _> 0.9 is always to the right of the curve. Panel (a): the reaction rates parameter space is projected on to (*K*_1_, *K*_2_) plane with *K*_3 _fixed at the values indicated in the panel. Panel (b): the parameter space is projected on the (*K*_2_, *K*_3_) plane with several choices for *K*_1 _as indicated in the panel.

Figure 5 shows similar kind of analysis as done for Figure 4 but for the first higher value of the Hill coefficient, *h *= 3. Unfortunately, because the structure of the parameter space is more complicated, it is not possible to use a single contour plot. Instead, various hyperplanes in the parameter space are studied. Panel (a) depicts the regions in the (*K*_1_, *K*_2_) plane where *δ*_max _= 0.9 for different choices of *K*_3_. The region with *δ*_max _> 0.9 is always to the right of each curve. For example, in the grey region in panel (a), for *K*_3 _= 1000, it is always true that *δ*_max _> 0.9. On the one hand, it can be seen that increase in *K*_3 _reduces the area where the fundamental similarity measure is large. On the other hand, for a fixed value of *K*_3_, and for a chemically functioning reactions (*K*_1_*K*_2 _~1), choosing *K*_1 _≫ *K*_2 _makes the fundamental similarity measure large. Likewise, panel (b) indicates that to obtain a large value for the fundamental similarity measure *K*_1 _should be as large as possible. For a given value of *K*_1 _one should take *K*_2 _≫ *K*_3_. In brief, one can say that *K*_1 _≫ *K*_2 _≫ *K*_3 _ensures that *δ*_max _is large but the plot shows that there are many subtle details associated with such a statement. Again, this confirms the previous finding in [5] that *K*_1 _≫ *K*_2 _≫ *K*_3 _results in similar dose response curves for both models, but please note that the statement made in here is much more general.

The quantitative analysis reveals rather rich structure of the parameter space where the two models have very similar noise characteristics (distribution functions). It would be useful to simplify such criteria. In that respect, it is tempting to express the strong-cooperativity criteria(10)

in another way, e.g. by introducing a measure of the degree of cooperativity *ξ *as(11)

The strong cooperativity can be characterized by *ξ *≫ 1. Naively, one would expect that in such a way one should obtain high values for *δ*_max _uniformly in *K*_1_.

**Figure 6:**
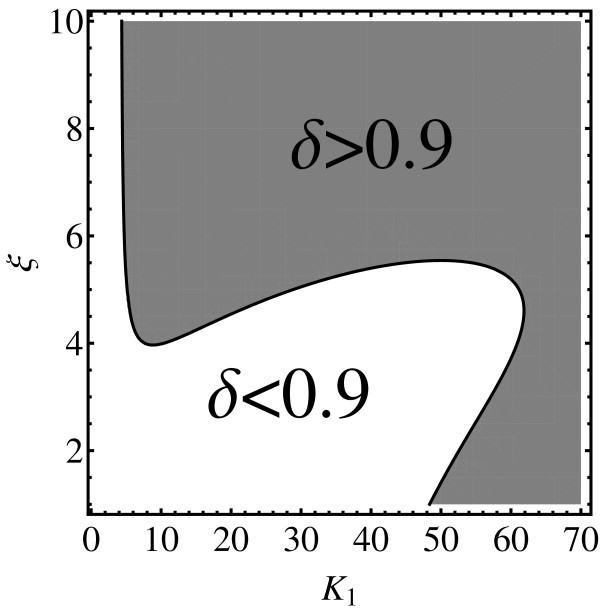
**Validity region of a *K*_1 _≫ *K*_2 _≫ *K*_3 _≫ *K*_4 _parameterization**. The plots depicts the boundary of the *δ*_max_(*K*_1_, *K*_2_, *K*_3_, *K*_4_) > 0.9 region in (*K*_1_, *ξ*) plane with the parameterization *K*_2 _= *K*_1_/*ξ*, *K*_3 _= *K*_1_/*ξ*^2^, and *K*_4 _= *K*_1_/*ξ*^3^. (*K*_0 _has been optimized as in the previous figures.)

Figure 6 is a contour plot that depicts how *δ*_max _depends on *K*_1 _and *ξ *for *h *= 4. The figure shows that many parameter choices that are chemically interesting do lead to a high value of the fundamental similarity measure (the grey region in the plot). Since there is no upper limit for *ξ*, for any value of *K*_1_, it is possible to choose *ξ *so that the reaction is chemically operational: for large *ξ *the product  becomes very small. However, there is rather large region close to the origin (the white region in the plot) where the Hill model is not a good replacement for the Adair-Klotz model. The minimal value of *ξ *that guarantees a good match needs to be adjusted depending on a value of *K*_1_. Interestingly, for *K*_1 _≳ 65 any value of *ξ *will lead to large *δ*_max_. Unfortunately, it was not possible to generate similar figures for *h *≥ 5 owing to the limitations of the computer hardware.

## Conclusions

Particle number fluctuations as predicted by Hill's and Adair-Klotz's model have been studied quantitatively. To compare the fluctuation characteristics of the two models, the similarity between the particle number distribution functions was characterized by three quantitative measures of similarity. The fundamental similarity measure *δ(t*) expresses the degree of overlap between the distribution functions on the common state space. Two auxiliary similarity measures  and  have been introduced to refine the analysis further by measuring the degree of occupancy of intermediate states, and measuring the similarity in the shape of the distributions on the common set of states.

It was shown that the similarity measures work as expected by studying their time dependence. The value of *δ(t*) always follows . This quantifies the intuitive expectation that the occupancy of the intermediate states governs whether models behave in the same way. In addition, it was found that, interestingly,  stayed relatively close to one, even when *δ*(*t*) was relatively small.

Furthermore, the equilibrium similarity measure *δ*_∞ _= lim_*t*→∞ _*δ*(*t*) was analyzed, where dependence of *δ*_∞ _on values of the dissociation constants *K*_1_, *K*_2_, ..., *K_h _*was carefully investigated. The analysis revealed that a value of the similarity measure in the equilibrium state is high when *K*_1 _≫ *K*_2 _≫ ... ≫ *K_h_*. This is in agreement with findings in an earlier work [5], which showed that the dose response curves for both models agree in this regime, provided the condition on the dissociation constants holds.

This work extends previous findings by avoiding the mean field approximation, and focussing on the distribution functions. By doing so it is possible to extend the previous finding to any property of interest that can be obtained from the particle number distribution functions. Furthermore, it was shown that the boundaries of the parameter space where *δ*_∞ _is high have a rather rich structure. While it is true that the condition *K*_1_≫ *K*_2_≫ ... *K_h _*guarantees that a given Adair-Klotz model can be substituted by a Hill's model, there are subtle details that need to be attached to such a statement.

The findings of this work should shed some light on the applicability of the previous uses of Hill's model. For example, Hill-like models have been used in the past to study characteristics of fluctuations in particle numbers during the process of complex formation [14-16]. This study shows that findings in these studies can be extrapolated to more realistic reaction models of complex formation, without doing the advanced technical analysis required for understanding more realistic reaction models.

This work can be extended in many ways. First, it should be possible to consider more challenging limits, with larger values of the Hill coefficient and particle copy numbers. Relatively small values for these parameters were considered owing to the limitations of the computer hardware (memory and CPU). Likewise, only pure states were considered, and it would be interesting to see whether the same conclusions can be drawn for other types of initial conditions. Second, instead of analyzing the full distribution functions, it should be possible to investigate the similarity of the underlying moments, and to define similarity measures accordingly. This could be advantageous for studying the problematic limits discussed above. Third, the similarity with, and among, other reaction models could be studied in a way similar to that presented here. For example, the issue of model reduction is a perpetual everlasting problem in the modelling of intracellular processes.

## Competing interests

The authors declare that they have no competing interests.

## Methods

### Computation of the distribution functions

To compare the models the particle number distribution functions will be investigated. It will be assumed that particles mix well. In such a setup, it is sufficient to count the particles. The numbers of *C*_0_, *C*_1_, *C*_2_, ..., *C_h _*and *A *particles will be denoted by *n*_0_, *n*_1_, *n*_2_, ..., *n_h _*and *n_A _*respectively.

Each system has a configuration space associated with it. The configuration spaces of the system are similar but not identical. For Hill's model a configuration of the system is given by *c_H _*= (*n*_0_, *n_h_*, *n_A_*), while for Adair-Klotz's model *c_A _*= (*n*_0_, *n*_1_, *n*_2_, ..., *n_h_*, *n_A_*). The difference comes from the fact that molecules *C*_1_, *C*_2_, ..., *C*_*h*-1 _need to be counted. In the following these molecules will be referred to as the intermediate molecules or, in brief, the intermediates.

The systems are stochastic and in course of time transitions within the configuration spaces of the systems occur randomly. The rapidity of transitions is governed by the previously introduced reaction rates. Both systems can be described by their respective master equations.

The master equation for Hill's model is given by(12)

where ∂*_t _*denotes the time derivative. The states *c_H_*[+,-, +] and *c_H_*[-,+,-] are defined by.(13)

where any combination of the plus and the minus signs can be picked at will (a choice has be to made consistently by picking either all upper or all lower signs). The particle number distribution function *P_H_*(*c_H_*, *t*) defines the occupancy probability for a state *c_H _*at a time *t*.

The master equation for Adair-Klotz's model is given by(14)

where(15)

where either the upper or the lower set of signs can be picked at will.

By solving the master equations (12) and (14) it is possible to obtain the distribution functions *P_H _*and *P_A _*for Hill's and Adair-Klotz's models respectively. In the next subsection the procedure for comparing the distributions will be discussed.

### Structure of the configuration spaces

To make a fair comparison between the models it is natural to use the same initial conditions for both. Since Hill's model does not have information about the intermediates, the initial conditions will be chosen so that the copy numbers of the intermediate species are all zero.

For Hill's model the dynamics will be started from a pure state with initial configuration given by(16)

where *P*_0 _and *L*_0 _denote the number of protein complexes and the number of ligand molecules in the system at *t *= 0. Likewise, for Adair-Klotz's model, the system will be started from(17)

For the pure initial state the dynamics of Hill's model occurs on the one dimensional space defined by the following states(18)

where  and the upper limit for the state index *i *is given by . The initial state corresponds to *i *= 0. This set of states will be referred to as(19)

Likewise, for a pure initial state, following set of states emerge for the Adair-Klotz model,(20)

Such set of states will be referred to as the Adair-Klotz space and denoted by(21)

where symbol _* _in the equation indicates that the upper limit has to be chosen such that occupancy numbers for each configuration are positive. Equation (20) indicates that protein molecules are either free from ligands, or have one or more ligands attached to them. From the perspective of the ligands, the equation states that all ligands that are not free are bound to protein molecules either as a single molecule, or in pairs, triples etc.

The inspection of the configurations for Hill's and Adair-Klotz'vs models, in (18) and (20), reveals that the configuration spaces are rather similar, up to the fact that the Adair-Klotz space has much higher rank.

Furthermore, it is possible to see that a vector in Adair-Klotz space with *i*_1 _= 0, *i*_2 _= 0, ..., *i*_*h*-1 _= 0 (Eq. 20) has a natural correspondence with the vector in the Hill space with *i *= *i_h _*(Eq. 18). In what follows it will be useful to formalize this mapping.

Symbol ℐ*_A_*(*c_H_*) will denote the image of a state *c_H _*in the Adair-Klotz space,(22)

The set of images of all vectors in the Hill space will be denoted by(23)

Please note that this mapping defines a one to one correspondence between the states in the Hill and the Adair-Klotz spaces. For example, given that *i *and *h *are fixed, there is only one combination of *i*_1_, ... *i_h _*for which *i *= *i*_1 _+ *i*_2 _+ ...+ *i_h _*and *hi *= *i*_1 _+ 2*i_2 _*...+ *hi_h_*.

Clearly, , and the set of states that are in the Adair-Klotz space but not in the image space (i.e. a complement) will be denoted by .

### Comparison of the distribution functions

To compare the probability distributions for the models, the distribution function for Adair-Klotz's model will be projected on to the state space of Hill's model:(24)

The direct comparison of *P_H _*(*c_H_*) with  can reveal whether there is a region in the parameter spaces of the two models where the respective dynamical behaviour is similar.

Once the projection is done, the comparison of the distribution functions is equivalent to the comparison of two vectors in a Cartesian space. For example, it is possible to use the scalar product between the vectors to compare them. However, for the purpose of this work, the distributions will be compared using(25)

The advantage of the particular form used in (25) is that for the perfect match with  for all *c_H _*∈ *S_H_*, the similarity measure *δ*(*t*) equals one. This can be seen from that fact that the sum in (25) becomes the normalization condition for the distribution functions. The lowest value for *δ*(*t*) is clearly zero since the distribution functions are positive definite. Also, please note that in the light of (16) and (17), *δ*(0) = 1. The initial conditions are chosen so that the match is perfect at *t *= 0. In such a way, any discrepancy detected by *δ*(*t*) is due to the dynamics of the systems.

### Fine tuning the comparison procedure

In addition to the similarity measure defined in Eq. (25) it is useful to analyze the extent to which the states in the complementary space  are populated. In that respect, it is useful to introduce(26)

This measure is important since it indicates to what extent the presence of intermediates affects the value of *δ*(*t*) in (25).

If the intermediate states are short lived, they should not be populated, and accordingly . In such a case *δ*(*t*) has a fair chance of being equal to one. On the other hand, for , *δ*(*t*) will be small, although the fact that the shapes of Hill's model distribution and Adair-Klotz's model distribution (projected on *S_H _*space) might be similar.

To analyze quantitatively the effects discussed above, it is useful to introduce a measure of the similarity of Hill's model distribution function and the normalized distribution function of Adair-Klotz's model  on Hill's space. To do this, it is useful to renormalize Adair-Klotz's model distribution function on the image space as(27)

where the norm is given by(28)

Please note that since Adair-Klotz's model distribution function is normalized, the following condition holds(29)

The similarity measure of Hill's model distribution function *P_H _*and the renormalized distribution function of Adair-Klotz's model  can be finally defined as(30)

Please note that  measures the similarity in the shapes of the distribution functions constrained on the Hill space, and in this work is referred to as the shape similarity measure.

Finally, using the equations above, it is trivial to show that(31)

The similarity of distributions can be factored in two contributions. The square root term on the right hand side of the equation measures the extent to which the image of the Hill space is populated for Adair-Klotz's model. The second term on the right hand side of the equation measures the similarity of the shape of the probability distributions on Hill's space image. To obtain a good match, both factors in the product need to be large, the intermediates should be short lived, and the shape of the distributions should be similar.

### Numerical computation setup

The distribution functions were computed by *Mathematica *using the technique of the Laplace transform. The Laplace transform of a function *f*(*t*) is defined in the usual way as(32)

The Laplace transform of the time derivative becomes an algebraic expression. Using this property, a master equation can be converted into an algebraic equation. The resulting linear algebraic equations were solved using the internal *Mathematica *solver. The asymptotic time limits of time-dependent functions were computed easily using(33)

Accordingly, the equilibrium quantities were computed with infinite precision.

For the time dependent quantities, the numerical inversion of the Laplace transform for the distribution functions was done using the Durbin method. The computations were performed using the *Mathematica *package developed by Arnaud Mallet and can be found at the repository of Mathematica packages. Thus the numerical results shown in the figures for time dependent quantities are exact to the accuracy of the numerical inversion procedure. The inversion formula is based on an integral that needs to be evaluated numerically. The accuracy of the result depends on the number of points used to perform the integral. This number was doubled incrementally until the relative change in the computed value was below 1%.
